# A new likelihood model for analyses of pharmacoepidemiologic case–control studies which avoids decision rules for determining latent exposure status

**DOI:** 10.1186/s12874-021-01312-y

**Published:** 2021-07-08

**Authors:** Henrik Støvring, Anton Pottegård, Jesper Hallas

**Affiliations:** 1 Biostatistics, Department of Public Health, Aarhus University, Bartholins Allé 2, 8000 Aarhus C, Denmark; 2 Clinical Pharmacology and Pharmacy, Department of Public Health, University of Southern Denmark, Odense, Denmark

**Keywords:** Case–control study, Reverse waiting time distribution, Maximum likelihood, Parametric modelling, Pharmacoepidemiology

## Abstract

**Background:**

Case–control studies based on pharmaco-epidemiological databases typically use decision rules to determine exposure status from information on dates of prescription redemptions, although this induces misclassification. The reverse Waiting Time Distribution has been suggested as a likelihood based model to estimate the latent exposure status, and we therefore suggest to extend this into a joint likelihood based model, which incorporates both the latent exposure status and the exposure-outcome association. This will achieve consistency and efficiency of the estimates, i.e. they can be expected to be asymptotically unbiased and have optimal precision.

**Methods:**

We established a joint likelihood for the observed case–control status and last prescription redemption before the index date. The likelihood combines the ordinary logistic regression likelihood and the reverse Waiting Time Distribution, and allows inclusion of covariates in both parts to adjust for observed confounders. We conducted a simulation study of the new model and standard models based on decision rules for exposure and the probability of being exposed, respectively, to assess the relative bias and variability of estimates. Lastly, we applied the models to a case–control study on use of nonsteroidal anti-inflammatory drugs and risk of upper-gastrointestinal bleeding.

**Results:**

In simulation studies the new model had low relative bias (< 1.4%) and largely retained nominal coverage probabilities (90.2% to 95.1% of nominal 95% confidence intervals), also when moderate misspecification was introduced. All standard methods generally had substantial bias (-21.1% to 17.0%) and low coverage probabilities (0.0% to 68.9%). When analyzing the empirical case–control study, the new method estimated the effect of nonsteroidal anti-inflammatory drugs on risk of with upper-gastrointestinal bleeding hospitalization to 2.52 (1.59 – 3.45), whereas the other methods had estimates ranging from 3.52 (2.19 – 5.65) to 5.17 (2.40 – 11.11).

**Conclusions:**

Unlike standard methods, the proposed model gave nearly unbiased estimates with adequate coverage probabilities in simulation studies. The developed model demonstrates the potential for the reverse Waiting Time Distribution to be integrated with existing likelihood based analyses in pharmacoepidemiological studies.

**Supplementary Information:**

The online version contains supplementary material available at 10.1186/s12874-021-01312-y.

## Background

Pharmacoepidemiologic case–control studies are commonly based on routinely collected register data, as this is an efficient strategy for assessing the risk of adverse drug effects in real-world settings. Typically in such studies, exposure information is obtained from databases recording all individual prescription redemptions for a population and then linked with information on for example hospital admissions for each individual in the population. Since the actual treatment after redeeming a prescription is not recorded, such studies routinely rely on decision rules for defining drug exposure status at the index time for both cases and controls. To avoid using such decision rules, we have previously developed the reverse Waiting Time Distribution (WTD) approach to estimate treatment duration after redeeming a prescription. In simulation studies and an empirical validation study for Warfarin, the method provided estimates with low bias, unless the model was severely misspecified [1, 2]. In a recent study, we suggested to use the reverse WTD to estimate the probability of being exposed on the index date, and use this probability as exposure variable [3]. The objective was to better allow for the fact that the actual exposure status is not recorded, and that the analyst may therefore introduce misclassification bias when forcing a dichotomy upon exposure. The approach was intended to directly represent the analyst’s uncertainty about actual exposure by using probability of being exposed as exposure covariate. We found that the approach improved statistical efficiency when studying the risk of upper gastrointestinal bleeding (UGIB) associated with use of a nonsteroidal anti-inflammatory drug (NSAID) [3]. The suggested method consisted of two steps: First, a parametric reverse WTD model was fitted to the last observed NSAID prescription redemption among controls before their index date to estimate their probability of being users on the index date. Second, we used the predicted probability as covariate in a logistic regression with case–control status as outcome.

However, both the traditional approach based on a simple decision rule and the approach using the probability of exposure as covariate are susceptible to misclassification bias. Also, they are not able to incorporate the uncertainty regarding the actual exposure into the subsequent analysis estimating the effect of being exposed with respect to the outcome of interest. This will bias uncertainty estimates for estimated parameters. To overcome these biases, we here propose to base estimation on a joint parametric likelihood for the last observed prescription redemption in the year preceding the index date, if any, together with the observed case–control status. From a theoretical point of view, formulation of a full likelihood model is attractive as it provides estimates based on a coherent and transparent model of what is actually observed, i.e. the case–control status and the time of last redemption before the index date. Since the observed data are conceptually linked together via the unobserved (i.e. latent) exposure status, we can obtain estimates for the exposure-outcome relation by maximizing the likelihood. Based on general likelihood theory, such estimates can be expected to have optimal large sample properties [4]. Note that when no prescription redemption is observed in the year before the index date, we can safely assume that the person is unexposed, and thus such persons can also be readily included in the analysis.

In this paper, we first establish the new joint likelihood, which is a synthesis of a reverse WTD model and the ordinary logistic regression of exposure status among cases and controls. In a simulation study we then compare the performance of the new joint likelihood model with classic decision-rule based analyses, as well as the previous suggestion of a two-step model based on exposure probabilities, and we investigate the impact of misspecification. We finally apply the models to a Danish case–control study with data on NSAID prescription redemptions and hospitalization with UGIB.

## Methods

Consider a case–control study with pharmacoepidemiologic exposure data. The observations include an index date for each case and control and their outcome status on the index date (case–control status), which we denote by $Y=1$ for cases and $Y=0$ for controls. Prior to the index date we observe a last prescription before the index date for each individual, if the individual has at least one prescription in a time window of observation before the index date. We let $R$ denote the time from this last prescription redemption to the index date, and let $V$ denote whether a prescription redemption was observed ($V=1$) or not ($V=0$).

**Fig. 1:**
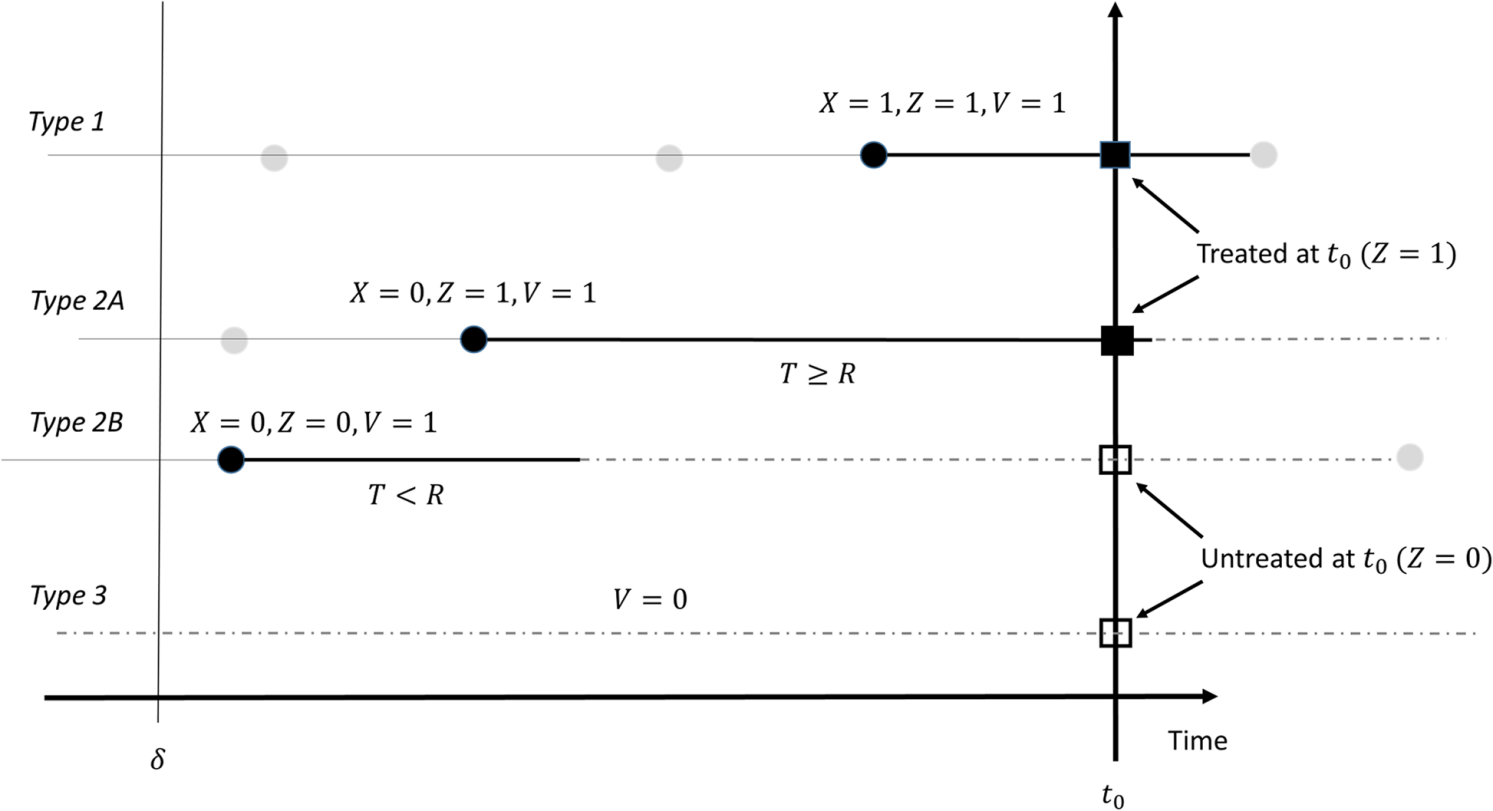
Four typical persons (horizontal lines) representing the different types of contributions to the likelihood with respect to exposure status on the index date indicated by squares (filled: treated, unfilled: untreated). Black bullets represent prescription redemptions included in the likelihood, grey bullets are redemptions not included in the likelihood. The thicker black lines represent periods of treatment, whereas dashed lines are periods without treatment. $T$ is duration of the prescription and $R$ is time from the last prescription redemption before the index date ${t}_{0}$ until the index date. $X$ indicates whether a patient continues treatment, $Z$ exposure status on the index date, and $V$ whether a prescription is redeemed in the interval $(\delta ;{t}_{0})$. For simplicity, index dates have been aligned on the time scale. Type refers to patient type, see text for details

As a starting point, let us assume that if a person is in continued treatment with a drug, then (s)he will redeem prescriptions following a renewal process with $f(t)$ as the inter-arrival density (IAD) between subsequent redemptions. When this renewal process is intercepted at an index date, $R$ is the time from the last renewal (redemption) before the index date until the index date (Fig. 1). We let $X$ denote whether a person continues having prescription redemptions ($X=1$) or not ($X=0$) after the last observed prescription redemption before the index date. Note that we have in previous papers shown that even if the assumption of redemptions following a renewal process is violated, then the backward recurrence time (time from last prescription redemption before the index date to the index date) can still be reasonably modelled as if originating from a renewal process [1, 5]. Also note, that since $X$ is not observed, we use this as a latent variable, whose distribution will depend on observed time since last prescription redemption and case–control status, as described below. This avoids conditioning on the future.

Concerning the outcome and exposure in the case–control study, a person can either be a case, $Y=1$, or a control, $Y=0$, at the index date ${t}_{0}$, and be exposed to treatment, $Z=1$, or not, $Z=0$. We will assume that the case–control status of a person depends on the true, but unobserved binary exposure status, $Z$, in the ordinary fashion of logistic regression, i.e.

$$P\left(Y=1|Z=z\right)=\frac{\mathrm{exp}\left({\beta }_{0}+{\beta }_{1}z\right)}{1+\mathrm{exp}\left({\beta }_{0}+{\beta }_{1}z\right)}$$

In this formulation, $\mathrm{exp}\left({\beta }_{1}\right)$ is the odds ratio of interest when studying the association between being exposed and becoming a case. To maintain mathematical feasibility, we will assume that if the true exposure status is known, then whether a person is a case or a control is unrelated to the distribution of time since last prescription redemption. In other words, we assume $Y$ and $R$ are independent given $Z$.

Let us now consider the relation between redemptions and exposure status. When a person continues treatment after the index date with one or more subsequent prescription redemptions ($$X=1$$ at the index date), then the person is by definition exposed at the index date ($$Z=1$$). However, when the last prescription redemption observed before the index date is also the last redemption in the treatment of an individual ($$X=0$$), such individuals can be either treated ($$Z=1)$$ or untreated ($$Z=0$$) on the index date (Fig. 1). This is because such individuals will remain exposed for some time after their last prescription redemption before they enter a state without treatment. We will assume that their duration of exposure after the prescription redemption, $$T$$, follows the ordinary IAD of treated patients continuing treatment, i.e. the probability density function of $$T$$ is $$f(t)$$. Finally, we will assume that persons without a prescription redemption in an interval before the index date, $$\left({t}_{0}-\delta ;{t}_{0}\right)$$, are unexposed. To accommodate this, we will let $$V$$ be an indicator variable, which is 1 if an individual has at least one redemption observed in $$({t}_{0}-\delta ;{t}_{0})$$ and 0 otherwise. Figure 1 gives an overview of the variables introduced and how they are related to four different characteristic patient types.

We can now write the likelihood contributions for each patient type by conditioning on $X,Z$, and $V$. In the following, we use $pdf\left(\cdot \right)$ as a generic probability density function for the relevant stochastic variables which are written as arguments. Patient type numbering refers to Fig. 1.

- *Patient type 1*: Patients continuing treatment, i.e. $X=1$, which implies that $Z = 1,V = 1$:
  $$\begin{array}{*{20}c} {pdf\left( {R = r,\left. {Y = y} \right|X = 1} \right) = pdf\left( {R = \left. r \right|X = 1} \right) \cdot pdf\left( {Y = \left. y \right|X = 1,R = r} \right)} \\ { = g\left( r \right)\frac{{\exp \left( {\beta _{0} + \beta _{1} } \right)^{y} }}{{1 + \exp \left( {\beta _{0} + \beta _{1} } \right)}}} \\ \end{array}$$
- *Patient type 2A and 2B*: Patients stopping treatment, i.e. $X = 0,V = 1$:
  $$\begin{array}{*{20}c} {pdf\left( {R = r,Y = \left. y \right|X = 0} \right)} \\ { = pdf\left( {Y = \left. y \right|Z = 0} \right) \cdot P\left( {Z = \left. 0 \right|R = r,X = 0} \right) \cdot pdf\left( {R = \left. r \right|X = 0} \right)} \\ { + pdf\left( {Y = \left. y \right|Z = 1} \right) \cdot P\left( {Z = \left. 1 \right|R = r,X = 0} \right) \cdot pdf\left( {R = \left. r \right|X = 0} \right)} \\ \begin{gathered} = \frac{1}{\delta }\left( {\frac{{\exp \left( {\beta _{0} } \right)^{y} }}{{1 + \exp \left( {\beta _{0} } \right)}}P\left( {Z = \left. 0 \right|R = r,X = 0} \right)} \right. \hfill \\ \left. { + \frac{{\exp \left( {\beta _{0} + \beta _{1} } \right)^{y} }}{{1 + \exp \left( {\beta _{0} + \beta _{1} } \right)}}P\left( {Z = \left. 1 \right|R = r,X = 0} \right)} \right) \hfill \\ \end{gathered} \\ \end{array}$$
  where $\frac{1}{\delta }$ is the uniform density associated with the last observed prescription redemption among patients stopping treatment. The uniform density corresponds to assuming a constant stopping rate with respect to the index date among treated. $P\left( {Z = \left. 1 \right|R = r,X = 0} \right) = 1 - F\left( r \right)$ is the probability of a person still being treated at the index date (patient type 2A), even though the person will stop treatment before any subsequent redemption. This is given by the probability of the treatment duration $T$ exceeding the time from the redemption to the index date, $r$, i.e.
  $$P\left(Z=1|R=r,X=0\right)=1-F\left(r\right)$$
  where $F\left(\cdot \right)$ is the cumulative density function of $T$. The density of $T$ is assumed to be the IAD of users continuing treatment, and from the definition of the BRD we have the following relation:
  $$g\left(r\right)=\frac{1-F\left(r\right)}{M}$$
  where $M$ is the mean prescription duration, $E(T)$. By definition this implies that the probability of being untreated at the index date for a patient stopping treatment (patient type 2B) is given by
  $$P\left(Z=0|R=r,X=0\right)=F\left(r\right)$$
- *Patient type 3*: Patients without an observed prescription redemption in the time window $\left({t}_{0}-\delta ;{t}_{0}\right)$, i.e. $V=0$, and who are therefore unexposed $\left(Z=0\right)$:
  $$pdf\left(Y=y|V=0\right)=\frac{{\mathrm{exp}\left({\beta }_{0}\right)}^{y}}{1+\mathrm{exp}\left({\beta }_{0}\right)}$$

To complete the likelihood, we sum the three components after multiplication with their probabilities of occurring, i.e. $P\left( {X = 1,V = 1} \right),P\left( {X = 0,V = 1} \right)$, and $P(V=0)$, respectively. With obvious notation, we can parametrize the probabilities as follows:

$$\begin{array}{*{20}l} {P\left( {X = 1,V = 1} \right) = P\left( {X = \left. 1 \right|V = 1} \right)P\left( {V = 1} \right) = p_{x} p_{v} } \hfill \\ {P\left( {X = 0,V = 1} \right) = P\left( {X = \left. 0 \right|V = 1} \right)P\left( {V = 1} \right) = \left( {1 - p_{x} } \right)p_{v} } \hfill \\ {P\left( {V = 0} \right) = \left( {1 - p_{v} } \right)} \hfill \\ \end{array}$$

In sum, the likelihood contribution for each individual becomes

$$\begin{array}{*{20}c} {l\left( {p_{v} ,p_{x} \beta _{0} ,\beta _{1} ,\left. \theta \right|r,y} \right) = p_{x} p_{v} \cdot g_{\theta } \left( r \right)\frac{{\exp \left( {\beta _{0} + \beta _{1} } \right)^{y} }}{{1 + \exp \left( {\beta _{0} + \beta _{1} } \right)}}} \\ { + \left( {1 - p_{x} } \right)p_{v} \frac{1}{\delta }\left( {\frac{{\exp \left( {\beta _{0} } \right)^{y} }}{{1 + \exp \left( {\beta _{0} } \right)}}F_{\theta } \left( r \right) + \frac{{\exp \left( {\beta _{0} + \beta _{1} } \right)^{y} }}{{1 + \exp \left( {\beta _{0} + \beta _{1} } \right)}}\left( {1 - F_{\theta } \left( r \right)} \right)} \right)} \\ { + \left( {1 - p_{v} } \right) \cdot \frac{{\exp \left( {\beta _{0} } \right)^{y} }}{{1 + \exp \left( {\beta _{0} } \right)}}} \\ \end{array}$$

The full likelihood for all individuals is the product of the individual likelihood contributions. We call this the joint likelihood as it is based on the joint density function for both the last prescription redemption before the index date, $R$, and the case–control status, $Y$. In the joint likelihood, $\theta$ denotes the parameters which the IAD, and thus the BRD, depends upon and which must be estimated. If we choose a Log-Normal distribution for $T$, then $\theta = \left( {\mu ,\sigma } \right)$, and we have the following expressions for ${g}_{\theta }$ and ${F}_{\theta }$:

$$\begin{array}{*{20}l} {g_{{\mu ,\sigma }} \left( r \right) = \frac{{\Phi \left( { - \frac{{\log \left( r \right) - \mu }}{\sigma }} \right)}}{{\exp \left( {\mu + \frac{1}{2}\sigma ^{2} } \right)}}} \hfill \\ {F_{{\mu ,\sigma }} \left( r \right) = \Phi \left( {\frac{{\log \left( r \right) - \mu }}{\sigma }} \right)} \hfill \\ \end{array}$$

where $\mathrm{\Phi }$ is the standard normal cumulative distribution function.

Note, that whether a case patient continues having prescription redemptions or not after the index date may be hypothetical in the sense that case status may prohibit any further treatment. The model does, however, only rely on the distribution of $R$ which is in principle unaffected by any termination of treatment after $Y=1$ has been observed (the case becomes a case).

Analogous with previous implementations of parametric WTDs, we have logit-transformed the probability parameters, ${p}_{x}$ and ${p}_{v}$, and log-transformed the parameter $\sigma$ which is required to be positive. Instead of a Log-Normal distribution, other parametric distributions with support on the positive axis could be considered, for example the Weibull or Gamma distributions.

We have previously shown how parameters of the parametric reverse WTD can be allowed to depend upon covariates in a regression-like setup [6], and the above formulation allows completely analogous inclusion of covariates. In particular, confounders which would ordinarily be included in an logistic regression analysis of the case–control study can be included in an equation for ${\beta }_{0}$. Also note that different parameters may depend on different sets of covariates.

### Simulation study

The aim of the simulation studies was to examine the bias and precision of estimates. In the simulation study of the finite sample properties, we generated data compatible with the model developed above. More specifically, we used the following steps to create each dataset:

1. Generate a dataset of a given size with the actual latent exposure to treatment and case/control status for each individual. The dataset must satisfy that there is a specific number of controls for each case (1 or 10), and such that the exposure status reflects a pre-specified odds-ratio value, here 3. Further, we specified the proportion of individuals expected to have a prescription redemption observed in the observation window of length one year (15% and 25%, i.e. values for the probability ${p}_{v}$ defined above), and how many of these that are expected to continue treatment after the index date (40 and 80%, i.e. values for ${p}_{x}$). The latter two proportions indicate the prevalence on the index date among individuals with a prescription redemption in the observation window. Based on these values it is possible to derive the probabilities of exposure among cases and controls, respectively, from which the exposure status can be drawn for all individuals in the dataset (see Additional file 1, Section A1 for details). Also for each individual it is randomly determined who has a prescription redemption in the observation window, and such that it is compatible with the random exposure status – exposed individuals must have a redemption in the observation window, whereas only some unexposed individuals will have such a redemption.
2. For the individuals assigned to continue treatment, and thus be exposed on the index date, we draw a prescription redemption before the index time, which follows a Log-Normal BRD. The parameters of the BRD, $\left( {\mu ,\sigma } \right)$, are chosen such that they correspond to IADs with a median duration of 1.5 or 2 months, i.e. $\mu = \log \left( {{{1.5} \mathord{\left/ {\vphantom {{1.5} {12}}} \right. \kern-\nulldelimiterspace} {12}}} \right)\;{\text{or}}\log \left( {{2 \mathord{\left/ {\vphantom {2 {12}}} \right. \kern-\nulldelimiterspace} {12}}} \right),\;{\text{and}}\;\sigma = \exp \left( { - 0.35} \right)\;{\text{or}}\;{\text{exp}}\left( { - 0.25} \right)$, respectively. The values for both parameters were similar in magnitude to estimates found in the analysis of the application presented below.
3. For individuals identified to stop treatment after index date, we randomly draw a uniformly distributed time of prescription redemption in the observation window, but such that for those still exposed on the index date their redemptions follow the above specified Log-Normal BRD. We used rejection sampling to satisfy the requirement of the marginal distribution being uniform among all treatment stoppers when redemptions of the exposed sub-population followed a Log-Normal BRD (see Additional file 1, Section A2 for details).

To examine the impact of a misspecified BRD, we also generated data with a Weibull BRD and analyzed the data with a model based on a Log-Normal BRD. To make the settings comparable, we used parameters such that the Weibull IAD had the same mean and variance as the Log-Normal IAD corresponding to the $\mu$ and $\sigma$ values stated above.

We generated datasets which consisted of 19,800 or 39,600 individuals as this either resembled the application below or had half the size of this application. For each scenario we generated 2,500 datasets, which we analyzed with five different approaches:

1. Logistic regression where exposure is the actually assigned exposure status, i.e. exposure is not latent and thus there is no misclassification bias.
2. The proposed joint likelihood model for case/control status and the rWTD, as developed above
3. Logistic regression with the estimated probability of being in treatment as exposure variable. The probability is estimated from an ordinary rWTD analysis.
4. Logistic regression where exposure status is based on a 90 days window – individuals are considered exposed if they have a prescription redemption within 90 days prior to their index date.
5. Logistic regression where exposure status is based on a 30 days window – individuals are considered exposed if they have a prescription redemption within 30 days prior to their index date.

All logistic regression analyses used case–control status as outcome. The first analysis is the reference analysis, as this is the analysis that would have been used for analyzing a case–control study with directly observed exposure status. It can be expected to yield unbiased estimates and have optimal statistical efficiency (smallest uncertainty intervals). We therefore benchmarked the other four analyses against it. Our primary interest was to establish whether the new joint model achieved its theoretical, asymptotic superiority in samples with a realistic size. For the two last approaches, we kept all generated individuals in the analysis to maintain comparability of standard errors with the other analyses, even though it is common practice in pharmacoepidemiologic studies to only include individuals which have no observed prescription redemptions (the reference group) or are classified as exposed (i.e. have a redemption inside the prespecified main exposure window) [3, 7].

For each method and setting we estimated the median relative bias of the estimated OR, the median standard error of the log-OR, the coverage probability of nominal 95% confidence intervals and finally the relative variance inflation factor with respect to the optimal analysis when exposure status is directly observed.

## Application

### Cases and controls

As case material we used a case–control study also employed in previous publications, which was described in detail there [3, 7]. Briefly, our source population was the residents of Funen County, Denmark, during a study period of August 1, 1995 to July 31, 2006. We included as cases all subjects who were admitted to a hospital on Funen with UGIB, validated by single case review.

Controls were selected by a risk-set sampling strategy, i.e. for each case, we randomly selected ten controls among the subjects in our source population who matched the case by sex and birth year. Controls were assigned an index date identical to the outcome defining date of the corresponding case. We required that both cases and controls had been residents of Funen for at least one year on the index date. As some of the very old cases had less than 10 eligible controls, the final control to case ratio deviated slightly from 10:1.

### Data analysis

Although data was collected with matching of controls to cases, we did not analyze the data conditional on match sets. Each matched set consisted of at most 11 individuals, which did not contain sufficient information to reliably estimate parameters of a WTD for each matched set. Consequently, we ignored the matching and instead included the matching variables age and sex as covariates in the association between exposure and case–control status, although this will likely introduce a small bias towards the null [8].

As reference analyses we considered logistic regression with case–control status as outcome and the exposure covariate with respect to NSAID defined as follows:

- WTD probability – the probability of being exposed as estimated by a reverse waiting time distribution among controls in the year prior to their index date. We let the WTD parameters depend on age, sex, quantity dispensed (in DDD), choice of ibuprofene (the dominant NSAID), concurrent use of proton pump inhibitors, a diagnosis of rheumatoid arthritis, psoriasis arthritis or spondylarthritis, and concurrent use of methotrexate or systemic corticosteroids. The latter variables (use of proton pump inhibitors and onwards) were intended to reflect markers of long-term NSAID use. Based on estimated parameters, observed covariates and date of last NSAID dispensing before the index date, we calculated the probability of being exposed on their index date for each case and control. This method mimics the approach used in a previous paper, where it was termed the *full multivariable model* [3].
- 90 days – exposed if at least one NSAID redemption was observed within 90 days before the index date
- 30 days – exposed if at least one NSAID redemption was observed within 30 days before the index date

For all the above models, we estimated a crude OR without adjustment for other variables and an adjusted OR. For the adjusted ORs, the following potential confounders were included as covariates in the logistic regression: 1) current use of the following drugs: vitamin K antagonists (VKA), aspirin, other antiplatelet drugs, dipyridamol, beta-blockers, selective serotonin reuptake inhibitors (SSRIs), systemic corticosteroids, proton pump inhibitors (PPIs), H2 receptor antagonists, statins, nitrates, spironolactone, calcium antagonists, bisphosphonates; 2) any history of the following events: NVUGB, *Helicobacter pylori* (HP) eradication, peptic ulcer, chronic obstructive pulmonary disease (COPD), diabetes, ischemic heart disease, heart failure, stroke, hypertension, inflammatory bowel disease, malignant disease, renal failure; and 3) prescription or diagnosis markers of smoking or excessive alcohol consumption. For all drugs used as covariates, current drug use was defined by the redeeming of a prescription within less than 120 days before the index date.

For the joint likelihood we let the parameters associated with the reverse WTD ($\mu ,\sigma$ and ${p}_{x}$) depend on the same covariates as the reverse WTD described above when using probability of exposure as covariate, i.e. the following variables: age, sex, quantity dispensed (in DDD), use of ibuprofene (the dominant NSAID), concurrent use of proton pump inhibitors, a diagnosis of rheumatoid arthritis, psoriasis arthritis or spondylarthritis, and concurrent use of methotrexate or systemic corticosteroids. For the logistic regression part of the joint likelihood we either omitted or included the same covariates (for ${\beta }_{0}$) as described above for the adjusted models. Correspondingly, we labeled the two models either crude or adjusted, although both included adjustment in the reverse WTD part of the model.

All statistical analyses were conducted in Stata 15 [9]. A dedicated software package (ccwtdttt) implementing the method is provided at the IDEAS repository (http://ideas.repec.org) and may be installed in Stata using a search for the package name, i.e. –search wtdttt, all–.

## Results

### Simulation study

Results of the simulation study are shown in Table 1 for the setting with a sample size of 39,600; a fraction of 80% of patients continuing treatment; 25% of all individuals having at least one prescription redemption in the time window of one year prior to the index date; and an OR of 3.

**Table 1:** Simulation results, data generated with a log-normal backward recurrence density

		**σ = exp(-0.35)**				**σ = exp(-0.25)**			
	**Analysis method**(*)	**Relative bias (%)**	**SE log(OR)**	**Coverage 95% CI (%)**	**VIF**	**Relative bias (%)**	**SE log(OR)**	**Coverage 95% CI (%)**	**VIF**
**μ = log(1.5/12)**	*1:1 CC*								
	**True expo**	0.0	0.027	95.6	1.00	0.0	0.027	95.3	1.00
	**CC WTD**	0.4	0.029	95.1	1.15	0.8	0.029	93.5	1.19
	**WTD prob**	17.0	0.037	0.0	1.91	17.1	0.038	0.0	2.00
	**90 days**	-6.7	0.028	24.3	1.08	-7.8	0.028	14.2	1.12
	**30 days**	-12.1	0.037	5.4	1.90	-12.9	0.038	3.8	2.01
	*1:10 CC*								
	**True expo**	0.0	0.036	95.1	1.00	0.0	0.036	94.5	1.00
	**CC WTD**	0.3	0.038	95.0	1.09	0.7	0.038	94.2	1.11
	**WTD prob**	8.7	0.046	46.4	1.61	8.3	0.047	51.4	1.66
	**90 days**	-7.5	0.038	40.0	1.06	-9.1	0.038	25.6	1.08
	**30 days**	-18.2	0.046	1.0	1.60	-19.2	0.047	0.7	1.69
**μ = log(2/12)**	*1:1 CC*								
	**True expo**	0.0	0.027	94.4	1.00	0.0	0.027	94.4	1.00
	**CC WTD**	1.0	0.029	93.6	1.19	1.7	0.030	90.2	1.24
	**WTD prob**	14.4	0.036	0.8	1.84	14.1	0.037	1.3	1.91
	**90 days**	-8.1	0.029	14.1	1.18	-9.2	0.030	7.6	1.23
	**30 days**	-13.9	0.041	3.8	2.35	-14.5	0.042	3.9	2.48
	*1:10 CC*								
	**True expo**	0.0	0.036	94.8	1.00	0.1	0.036	94.7	1.00
	**CC WTD**	0.8	0.038	94.6	1.11	1.4	0.039	93.6	1.14
	**WTD prob**	6.7	0.045	62.6	1.56	6.1	0.046	68.9	1.60
	**90 days**	-10.2	0.038	17.2	1.12	-11.7	0.039	8.2	1.15
	**30 days**	-21.1	0.051	0.5	1.94	-21.5	0.052	0.3	2.03

The datasets had a sample size of 39,600, and on average 80% of patients continue treatment at the index date, 25% of patients have a prescription redemption in the year before the index date and the true OR is 3. For each setting 2,500 datasets were generated and analyzed. $\mu$ and $\sigma$ are parameters of the assumed Log-Normal Backward Recurrence Density used for generating data, see text for details

(*) Analysis methods:

*1:1 CC* indicates 1 control per case, *1:10 CC* indicates 10 controls per case

**True expo** – logistic regression with the actual exposure status as covariate (the reference analysis)

**CC WTD** – estimation based on joint likelihood for case–control status and the reverse WTD, Log-Normal Backward Recurrence Density

**WTD prob** – a reverse WTD with Log-Normal Backward Recurrence Density is estimated to predict the probability of an individual being exposed and this exposure probability is used as covariate in logistic regression

**90 days** – individuals are considered exposed if they have a redemption < 90 days before index date

**30 days** – individuals are considered exposed if they have a redemption < 30 days before index date, logistic regression

The new method based on a joint likelihood for the case status and last observed prescription redemption before the index date was virtually unbiased (range 0.3 to 1.7%) with coverage probabilities close to the nominal value (range 90.2 to 95.1%) and relatively low VIFs (range 1.09 to 1.24). With 1:1 matching and a low $\mu$ value (median prescription duration of 1.5 months) there were a small number of datasets for which the likelihood estimation did not converge (< 70 of 2,500).

Results were largely similar for the joint likelihood method when the sample size was halved to 19,800, although with higher coverage probabilities (see Additional file 1, Table A1) or when the fraction of patients continuing treatment was reduced to 40% (see Additional file 1, Table A2). VIFs were however increased when fewer continued treatment (range 1.23 to 1.53). The other methods remained biased with low coverage probabilities, although their internal rank of performance changed in some settings.

**Table 2:** Simulation results, data generated with a Weibull backward recurrence density

		**σ = exp(-0.35)**				**σ = exp(-0.25)**			
	**Analysis method**(*)	**Relative bias (%)**	**SE log(OR)**	**Coverage 95% CI (%)**	**VIF**	**Relative bias (%)**	**SE log(OR)**	**Coverage 95% CI (%)**	**VIF**
**μ = log(1.5/12)**	*1:1 CC*								
	**True expo**	0.0	0.027	95.7	1.00	0.0	0.027	95.8	1.00
	**CC WTD**	0.9	0.029	94.2	1.15	0.5	0.029	95.1	1.18
	**WTD prob**	22.6	0.040	0.0	2.24	18.0	0.038	0.1	2.05
	**90 days**	-6.0	0.027	33.2	1.06	-8.1	0.028	11.2	1.13
	**30 days**	-11.1	0.035	6.7	1.69	-13.3	0.038	2.4	2.02
	*1:10 CC*								
	**True expo**	0.0	0.036	95.4	1.00	0.0	0.036	95.2	1.00
	**CC WTD**	0.6	0.038	94.9	1.08	0.5	0.038	95.2	1.10
	**WTD prob**	11.2	0.049	28.7	1.78	8.6	0.047	48.5	1.68
	**90 days**	-6.5	0.037	52.2	1.04	-9.5	0.038	20.6	1.09
	**30 days**	-16.8	0.044	1.1	1.45	-19.6	0.048	0.3	1.70
**μ = log(2/12)**	*1:1 CC*								
	**True expo**	-0.1	0.027	94.2	1.00	0.0	0.027	95.8	1.00
	**CC WTD**	1.7	0.029	90.1	1.21	1.6	0.030	91.5	1.25
	**WTD prob**	18.8	0.039	0.1	2.13	14.4	0.037	0.9	1.97
	**90 days**	-8.0	0.029	13.7	1.16	-10.2	0.030	3.5	1.26
	**30 days**	-13.4	0.038	3.4	2.09	-15.2	0.042	2.6	2.50
	*1:10 CC*								
	**True expo**	0.0	0.036	94.6	1.00	0.1	0.036	95.3	1.00
	**CC WTD**	1.4	0.038	92.9	1.11	1.7	0.039	92.7	1.14
	**WTD prob**	8.5	0.048	49.2	1.71	5.9	0.046	71.5	1.63
	**90 days**	-9.9	0.038	18.4	1.10	-12.8	0.039	4.7	1.17
	**30 days**	-20.0	0.048	0.4	1.74	-22.1	0.052	0.2	2.05

The datasets had a sample size of 39,600, and on average 80% of patients continue treatment at the index date, 25% of patients have a prescription redemption in the year before the index date and the true OR is 3. For each setting 2,500 datasets were generated and analyzed. The Weibull Backward Recurrence Density used to generate data corresponded to a Weibull distribution with the same mean and variance as a Log-Normal distribution with $\mu$ and $\sigma$ as its parameters, see text for details

(*) Analysis methods:

*1:1 CC* indicates 1 control per case, *1:10 CC* indicates 10 controls per case

**True expo** – logistic regression with the actual exposure status as covariate (the reference analysis)

**CC WTD** – estimation based on joint likelihood for case–control status and the reverse WTD, Log-Normal Backward Recurrence Density

**WTD prob** – a reverse WTD with Log-Normal Backward Recurrence Density is estimated to predict the probability of an individual being exposed and this exposure probability is used as covariate in logistic regression

**90 days** – individuals are considered exposed if they have a redemption < 90 days before index date

**30 days** – individuals are considered exposed if they have a redemption < 30 days before index date, logistic regression

In all settings there was substantial bias associated with the method of choosing a fixed exposure window, be it 30 or 90 days (relative bias ranged from -6.7 to -21.5%). Correspondingly, the coverage probabilities of nominal 95% confidence intervals for the odds ratio were low (range 0.3 to 40%). The variance inflation factor (VIF) varied considerably relative to the optimal analysis in which the true exposure status of individuals was directly observed (range 1.06 to 2.48), but given the substantial bias this is less relevant.

The analysis based on estimating the probability of exposure with the rWTD and then use this probability as exposure covariate also led to substantial relative bias (range 6.1 to 17.1%), which indicates that this approach in general overestimated the association. This approach also had low coverage probabilities (range 0.0 to 68.9%), but consistently it had high VIFs relative to the optimal analysis (range 1.56 to 2.00). The low coverage probabilities were thus not so much a consequence of small estimated standard errors, but rather of the magnitude of the bias.

When the BRD was misspecified (data generated from a Weibull BRD and analyzed with a model based on a Log-Normal BRD), the relative bias increased slightly for the joint likelihood ($\le$ 1.7%), coverage probabilities remained near the nominal level ($\ge$ 90.1%), and VIFs were as above ($\le$ 1.25) (Table 2). Similar results were seen with the smaller sample size of 19,800 (see Additional file 1, Table A3). The bias, coverage and VIFs for the other analytic approaches were similar to the settings where a Log-Normal BRD was used for generating the data.

**Table 3:** Estimated association between NSAID use and severe upper gastrointestinal bleeding

Exposure definition	*n*	Crude OR (95% confidence interval)	Adjusted OR (95% confidence interval)	Upper/lower confidence limit ratio for adjusted OR
Fixed window, 30 days	9,453			
*Conditional*		7.06 (6.17—8.06)	5.17 (2.40—11.11)	4.62
*Ordinary*		6.91 (6.19—7.71)	3.85 (2.09—7.07)	3.38
Fixed window, 90 days	12,662			
*Conditional*		4.96 (4.46—5.51)	4.73 (2.72—8.23)	3.02
*Ordinary*		4.87 (4.43—5.36)	3.52 (2.19—5.65)	2.58
WTD treatment probability	39,119			
*Conditional*		6.99 (6.35—7.69)	4.37 (3.62—5.28)	1.46
*Ordinary*		6.90 (6.28—7.58)	3.94 (3.29—4.72)	1.43
Joint likelihood model	39,119	5.57 (5.08 ‐ 6.05)	2.52 (1.59 – 3.45)	2.18

Case–control study of 3568 cases and 35,552 controls. See text for technical description of exposure definitions and covariate adjustment. *Conditional* results are based on conditional logistic regression which accounts for matching on age and sex. *Ordinary* results are based on ordinary logistic regression (unconditional), but where sex and age are included as covariates, both in the crude and adjusted analyses

### Application

The characteristics of cases and controls are shown in an additional table (see Additional file 1, Table A4). The joint likelihood model provided an unadjusted estimate of the NSAID effect on risk of UGIB similar to the other models (OR = 5.57 (5.08—6.05)), although the estimate based on the probability of being exposed was higher (Table 3). However, when the association was adjusted for confounders, the estimated effect (2.52 (1.59—3.45)) was substantially lower for the joint model than estimates from the three other models. The joint model had better precision than the methods based on a fixed exposure window, but lower than the exposure probability approach. As in the previous papers, when reporting estimates based on a fixed exposure window we omitted patients who had an NSAID prescription outside the exposure window resulting in a lower sample size for these two analyses (30 days: *n* = 9,453 and 90 days: *n* = 12,662 vs the full sample size of 39,119). The gain in precision for the models based on the WTD is thus due to their ability to include the entire sample in the estimation. Estimates were largely unchanged, whether we used conditional logistic regression to account for the age- and sex-matched design or included sex and age as covariates in ordinary logistic regression with all three standard analyses. However, for the adjusted analyses, estimated ORs were smaller for the two analyses based on a fixed exposure window (30 days or 90 days).

## Discussion

We have shown how it is possible to establish a likelihood for the effect of a latent exposure status in a case–control study, where the latent exposure status is modelled via a reverse Waiting Time Distribution (WTD). The reverse WTD relies on data regarding the last prescription redemption before the index date, if any, which are observed in pharmacoepidemiologic databases. Simulation studies showed that the developed model provided nearly unbiased estimates with confidence intervals largely achieving nominal coverage probabilities in settings with realistic sample sizes, even with a misspecified model. This was not the case for the two standard approaches with fixed exposure windows, nor the method based on using estimated exposure probability as covariate. The precision of the joint likelihood estimates was also better than the other methods as it had Variance Inflation Factors smaller than 1.25 relative to a model based on observing the true exposure status of all individuals. In the application, the joint likelihood model gave a lower adjusted estimate of the risk of UGIB associated with NSAID use than other methods. The joint likelihood model can be estimated with a Stata package we have made available online.

Theoretically, a full likelihood-based model provides the optimal analysis in terms of statistical efficiency, since it for large samples provides unbiased estimates with minimal standard errors (Cramér-Rao lower bound) [4]. Other methods may yield smaller standard errors, but are then not consistent as they are asymptotically biased. We therefore combined the likelihood of the reverse WTD and that of a logistic regression to allow likelihood estimation of the OR in case–control studies with exposure information based on registry data in a single joint model. We based our approach on noting that logistic regression in a case–control study models the relation between exposure and case–control status. When using pharmacoepidemiologic register data, exposure status is not directly observed (did the patient actually take the pill on the index date?), but this latent exposure status is modelled in the reverse WTD. The reverse WTD links information on last observed prescription redemption before the index date to two different latent sub-groups of patients: those who are continuing treatment on the index date and those who are stopping treatment. In our approach we allowed for the fact that patients, who have no dispensings after their last observed prescription redemption prior to the index date, may still be in treatment on that index date, as they use up the dispensed quantity. As expected, the model in simulation studies provided virtually unbiased estimates for realistic sample sizes with valid confidence intervals when analyzing data generated from the same model as that used for analysis, i.e. when the model was correctly specified. Since the model could not rely on observation of actual exposure status, the precision of its estimated effect was lower than for a corresponding model based on observed exposure status (VIFs ranged from 1.11 to 1.24 in simulations). It should be noted that since actual treatment status is a latent parameter in the model, it does not determine individual exposure status as such – rather it estimates the most credible parameters governing the exposure-outcome association in combination with parameters of the reverse WTD. For the other methods, it was somewhat surprising for us that the method using probability of exposure as covariate was not unbiased. This is however due to misspecification of exposure status: If exposure status is truly binary, as is the case in our simulation, then any estimated probability of exposure represents a misclassification of the individual’s exposure status, which will result in bias [10].

We are not aware of other methods that allow direct estimation of the exposure OR in a case–control study in which exposure status is latent and only indirectly linked to observed prescription redemptions. Abrahamowicz and co-authors investigated how different models for the exposure-outcome relation over time could be used in prospective studies on the risk of adverse effects with pharmacoepidemiological registry data [11]. They relied on model diagnostic criteria to identify the correct model among many candidates and found substantial bias associated with misspecification of the exposure-outcome relation with respect to timing. In our model we assumed that it was the exposure status on the index date, although latent, that induced a potential effect on case–control status.

The main advantage of our model is its reliance on general likelihood theory and an explicitly stated model for which assumptions can be checked. The model can therefore be expected to work well in a variety of settings irrespective of disease and outcome, as long as treatment consists of episodes with multiple prescription redemptions and that the relevant exposure is a binary status on the index dates for cases and controls. Still, the model also has several limitations. First, the method does not allow taking into account any matching. This is expected to dilute effect estimates [12]. Including matching variables as covariates in the estimation may to some extent remedy this, and we did indeed see that this strategy provided similar results with the standard analyses in our application. Second, even when the model is correctly specified it is complex, and the maximum likelihood estimation procedure may occasionally not converge. Third, the method relies on the simplifying assumptions of the BRD being similar between cases and controls, and that patients having no dispensings after the index date may remain in treatment for a period of time after their last prescription redemption with a distribution of prescription durations similar to that seen among patients continuing treatment. Fourth, while we did examine the method’s performance with some misspecification of the BRD, it is not clear how vulnerable the method is to more severe model-misspecification. For mathematical feasibility we modeled event times and case–control status as independent given the latent exposure status, but this may not hold for NSAID and UGIB, where we expect recent initiators to have a higher risk than later in the treatment phase [13]. Addressing this will require further modelling, but we have likely underestimated the effect of NSAID on UGIB in our analysis based on the developed joint likelihood. Finally, the developed model only estimates the effect of current use, i.e. the effect of being exposed on the index date (a binary covariate). In many settings, it will be of interest to allow for more complex characteristics of exposure such as time since treatment initiation and total amount consumed. This is however complex, and more work is needed to incorporate this in the model.

## Conclusions

We have developed a novel likelihood-based method which provides direct estimation of the exposure effect in case–control studies with exposure information obtained from pharmacoepidemiological data. The most important contribution is to show how formal extension of the reverse WTD model allows flexible estimation without use of expert input on expected patterns of drug use for definition of exposure status. Specifically, the established model provides estimates relying only on the actually observed data, i.e. case–control status and last prescription redemption before the index date, possibly supplemented with information on confounders that can be included in the estimation. The set of covariates included can potentially vary between parameters in the joint likelihood, such that for example the reverse WTD part of the model can rely on covariates informative for drug use patterns, while the effect parameter can be adjusted for confounding variables. The method can be expected to provide a similar precision to a study with observed exposure status without misclassification, if it has a 25–50% larger sample size. The developed model only applies to case–control studies, but may provide a starting point for development of models for other study designs.

## Supplementary Information

**Additional file 1**

## Data Availability

Due to Danish laws on personal data, data cannot be shared publicly. To request these data, please contact the Danish Health Data Authority.

## Abbreviations

BRD: Backward recurrence density
IAD: Inter-arrival density
NSAID: Non-steroidal anti-inflammatory drugs
OR: Odds ratio
UGIB: Upper gastrointestinal bleeding
VIF: Variance Inflation Factor
WTD: Waiting Time Distribution
