# Supplementary material for: A new likelihood model for analyses of pharmacoepidemiologic case–control studies which avoids decision rules for determining latent exposure status
Source: BMC Med Res Methodol. 2021 Jul 8;21:144. doi: 10.1186/s12874-021-01312-y (PMC8265059; doi:10.1186/s12874-021-01312-y)
Supplement: Supplementary file 1 — Additional file 1 [file 12874_2021_1312_MOESM1_ESM.docx]

# Additional material

## Section A1: Determining the proportion of exposed cases in simulations

In the simulation study we need to generate exposure status and case-control such that there is a pre-specified odds-ratio (OR), a pre-specified ratio of cases to controls, and a pre-specified fraction of exposed. The problem can be described as in the following Table:

| Case-control status | Exposed | Unexposed | Total |
| --- | --- | --- | --- |
| Case | $p_{11}$ | $p_{10}$ | $p_{1}$ |
| Control | $p_{01}$ | $p_{00}$ | $(1-p_{1})$ |
| Total | $\pi_{1}$ | $(1-\pi_{1})$ | 1 |

Note, that we do not condition on there being an exact ratio of cases to controls, nor on a fixed ratio of exposed to unexposed. Our objective is that we want to provide values for the OR, $p_{1}$ and $\pi_{1}$ and then obtain values of the four $p_{ij}$’s.

The OR can be written as

$$OR=\frac{p_{11}\cdot p_{00}}{p_{01}\cdot p_{10}}$$

We want to isolate $p_{11}$, and we therefore rewrite it as follows (note: $p_{01}=\pi_{1}-p_{11}; p_{10}=p_{1}-p_{11};$ and $p_{00}=1-\pi_{1}-p_{1}+p_{11}$):

$$OR=\frac{p_{11}\cdot\left( 1-\pi_{1}-p_{1}+p_{11} \right)}{(\pi_{1}-p_{11})(p_{1}-p_{11})}$$

We can now find $p_{11}$ by solving this equation, which corresponds to finding solutions to the following second order equation:

$$\left( OR-1 \right)p_{11}^{2}+\left( \pi_{1}+p_{1}-1-OR\left( \pi_{1}+p_{1} \right) \right)p_{11}+OR\cdot\pi_{1}\cdot p_{1}=0$$

There are in principle two solutions to this equation, but only the following is relevant:

$$p_{11}=\frac{-B-\sqrt{B^{2}-4AC}}{2A}$$

Where $A, B$ and $C$ are given as:

$$A=\left( OR-1 \right)$$

$$B=\left( \pi_{1}+p_{1}-1-OR\left( \pi_{1}+p_{1} \right) \right)$$

$$C=OR\cdot\pi_{1}\cdot p_{1}$$

With values of $p_{11}$, $\pi_{1}$ and $p_{1}$ it is straightforward to find the values of $p_{01}, p_{10}$ and $p_{00}$.

The final step in the generation of exposure statuses is to note that the overall proportion of exposed, $\pi_{1}$, is given by the proportion of subjects with a redemption within the year preceding the index date, $p_{v}$, times the conditional probability of being exposed among these at the index date, $P(Z=1|V=1)$. The latter probability is given by

$$P\left( Z=1 \right|V=1)=P\left( Z=1,X=1 | V=1 \right)+P(Z=1, X=0|V=1)$$

$$=p_{x}+P\left( T>R|X=0, V=1 \right)\left( 1-p_{x} \right)$$

The probability $P\left( T>R|X=0, V=1 \right)$ was obtained from numerical integration of the cumulative distribution function for the Log-Normal with parameters $(\mu, \sigma)$, since $T$ is assumed to follow a Log-Normal distribution and $R$ is uniformly distributed among patients with an observed prescription redemption who are stopping treatment.

## Section A2: Obtaining a uniform marginal when a certain fraction of stopping users are exposed on the index date

When exposure status $Z$ has been generated for all individuals in a simulated dataset, we need to generate the time of the last prescription redemption before the index date for all individuals who have been assigned to have such a prescription redemption $\left( V=1 \right)$. This consists in drawing from the relevant BRD for all individuals who are exposed $(Z=1)$, and supplement with draws of redemption times for unexposed individuals stopping treatment, such that the marginal distribution of redemption times among exposed stoppers and unexposed stoppers becomes uniform. For a Log-Normal BRD we achieve this by rejection sampling, where we keep the uniform draw of a prescription time $U_{1}$ for an unexposed stopper, if $U_{1}$ satisfies the following inequality:

$$U<\frac{\Phi\left( \frac{\left( \log\left( U_{1} \right)-\mu\right)}{\sigma} \right)}{\Phi\left( \frac{\left( \log\left( \delta\right)-\mu\right)}{\sigma} \right)}$$

Here $\Phi(\cdot)$ is the standard normal cumulative distribution function, $(\mu,\sigma)$ are parameters of the Log-Normal distribution used for the IAD, and $U$ is uniform random variate. $\delta$ is the width of the time window in which prescription redemptions are included (one year). A similar approach can be used with distributions for the BRD, such as for example the Weibull, also in simulations.

Table A1. Simulation results for the setting with data generated from a Log-Normal Backward Recurrence Density (BRD) and analyzed with a Log-Normal BRD. The datasets had a sample size of 19,800, and on average 80% of patients continue treatment at the index date, 25% of patients have a prescription redemption in the year before the index date and the true OR is 3. For each setting 2,500 datasets were generated and analyzed. $\mu$ and $\sigma$ are parameters of the assumed Log-Normal BRD used for generating data, see text for details.

|  |  | **σ=exp(-0.35)** | | | |  | **σ=exp(-0.25)** | | | |
| --- | --- | --- | --- | --- | --- | --- | --- | --- | --- | --- |
|  | **Analysis method** *(*)* | **Relative bias (%)** | **SE log(OR)** | **Coverage 95% CI (%)** | **VIF** |  | **Relative bias (%)** | **SE log(OR)** | **Coverage 95% CI (%)** | **VIF** |
| **μ=log(1.5/12)** | *1:1 CC* |  |  |  |  |  |  |  |  |  |
|  | **True expo** | -0.1 | 0.038 | 94.9 | 1.00 |  | 0.0 | 0.038 | 94.9 | 1.00 |
|  | **CC WTD** | 0.3 | 0.041 | 94.8 | 1.15 |  | 0.9 | 0.041 | 94.2 | 1.19 |
|  | **WTD prob** | 16.9 | 0.052 | 5.7 | 1.92 |  | 16.9 | 0.053 | 6.3 | 1.99 |
|  | **90 days** | -6.8 | 0.039 | 52.3 | 1.08 |  | -7.8 | 0.040 | 42.1 | 1.12 |
|  | **30 days** | -12.1 | 0.052 | 27.1 | 1.89 |  | -12.8 | 0.054 | 25.3 | 2.01 |
|  | *1:10 CC* |  |  |  |  |  |  |  |  |  |
|  | **True expo** | -0.1 | 0.052 | 94.5 | 1.00 |  | 0.0 | 0.052 | 94.1 | 1.00 |
|  | **CC WTD** | 0.3 | 0.054 | 94.4 | 1.09 |  | 0.8 | 0.054 | 94.4 | 1.11 |
|  | **WTD prob** | 8.5 | 0.066 | 70.5 | 1.61 |  | 8.3 | 0.066 | 72.4 | 1.66 |
|  | **90 days** | -7.6 | 0.053 | 65.7 | 1.06 |  | -9.0 | 0.054 | 54.3 | 1.08 |
|  | **30 days** | -18.3 | 0.065 | 13.5 | 1.60 |  | -19.1 | 0.067 | 11.5 | 1.69 |
|  |  |  |  |  |  |  |  |  |  |  |
| **μ=log(2/12)** | *1:1 CC* |  |  |  |  |  |  |  |  |  |
|  | **True expo** | 0.1 | 0.038 | 94.7 | 1.00 |  | 0.0 | 0.038 | 95.1 | 1.00 |
|  | **CC WTD** | 1.1 | 0.041 | 94.3 | 1.19 |  | 1.6 | 0.042 | 93.5 | 1.24 |
|  | **WTD prob** | 14.4 | 0.051 | 13.1 | 1.84 |  | 13.9 | 0.052 | 15.6 | 1.91 |
|  | **90 days** | -7.9 | 0.041 | 43.9 | 1.18 |  | -9.2 | 0.042 | 33.3 | 1.23 |
|  | **30 days** | -14.0 | 0.058 | 25.7 | 2.36 |  | -14.5 | 0.059 | 23.6 | 2.49 |
|  | *1:10 CC* |  |  |  |  |  |  |  |  |  |
|  | **True expo** | 0.2 | 0.051 | 94.7 | 1.00 |  | -0.1 | 0.051 | 95.4 | 1.00 |
|  | **CC WTD** | 0.9 | 0.054 | 94.4 | 1.11 |  | 1.3 | 0.055 | 94.6 | 1.14 |
|  | **WTD prob** | 6.8 | 0.064 | 78.4 | 1.56 |  | 5.8 | 0.065 | 82.4 | 1.60 |
|  | **90 days** | -10.1 | 0.054 | 46.2 | 1.12 |  | -11.8 | 0.055 | 34.9 | 1.15 |
|  | **30 days** | -21.0 | 0.072 | 10.2 | 1.93 |  | -22.0 | 0.073 | 7.7 | 2.03 |

*(*) Analysis methods:*

*1:1 CC* indicates 1 control per case, *1:10 CC* indicates 10 controls per case.

**True expo** – logistic regression with the actual exposure status as covariate (the reference analysis).

**CC WTD** – estimation based on joint likelihood for case-control status and the reverse WTD.

**WTD prob** – a reverse WTD is estimated to predict the probability of an individual being exposed and this exposure probability is used as covariate in logistic regression.

**90 days** – individuals are considered exposed if they have a redemption < 90 days before index date.

**30 days** – individuals are considered exposed if they have a redemption < 30 days before index date, logistic regression.

Table A2. Simulation results for the setting with data generated from a Log-Normal Backward Recurrence Density (BRD) and analyzed with a Log-Normal BRD. The datasets had a sample size of 39,600, and on average 40% of patients continue treatment at the index date, 25% of patients have a prescription redemption in the year before the index date and the true OR is 3. For each setting 2,500 datasets were generated and analyzed. $\mu$ and $\sigma$ are parameters of the assumed Log-Normal BRD used for generating data, see text for details.

|  |  | **σ=exp(-0.35)** | | | |  | **σ=exp(-0.25)** | | | |
| --- | --- | --- | --- | --- | --- | --- | --- | --- | --- | --- |
|  | **Analysis method** *(*)* | **Relative bias (%)** | **SE log(OR)** | **Coverage 95% CI (%)** | **VIF** |  | **Relative bias (%)** | **SE log(OR)** | **Coverage 95% CI (%)** | **VIF** |
| **μ=log(1.5/12)** | *1:1 CC* |  |  |  |  |  |  |  |  |  |
|  | **True expo** | -0.1 | 0.034 | 94.8 | 1.00 |  | 0.1 | 0.034 | 95.1 | 1.00 |
|  | **CC WTD** | -0.5 | 0.040 | 95.0 | 1.39 |  | -0.5 | 0.041 | 94.4 | 1.46 |
|  | **WTD prob** | 12.9 | 0.045 | 17.6 | 1.83 |  | 12.5 | 0.046 | 21.0 | 1.91 |
|  | **90 days** | -17.0 | 0.033 | 0.0 | 0.95 |  | -17.8 | 0.033 | 0.0 | 0.98 |
|  | **30 days** | -8.6 | 0.047 | 47.9 | 1.96 |  | -9.5 | 0.048 | 42.4 | 2.07 |
|  | *1:10 CC* |  |  |  |  |  |  |  |  |  |
|  | **True expo** | 0.0 | 0.041 | 95.3 | 1.00 |  | -0.1 | 0.041 | 95.3 | 1.00 |
|  | **CC WTD** | -0.2 | 0.046 | 95.2 | 1.23 |  | -0.3 | 0.046 | 94.6 | 1.27 |
|  | **WTD prob** | 10.2 | 0.054 | 44.6 | 1.71 |  | 9.5 | 0.055 | 51.8 | 1.78 |
|  | **90 days** | -12.6 | 0.042 | 8.1 | 1.05 |  | -13.9 | 0.043 | 5.0 | 1.08 |
|  | **30 days** | -11.9 | 0.055 | 34.6 | 1.81 |  | -13.0 | 0.057 | 28.1 | 1.91 |
|  |  |  |  |  |  |  |  |  |  |  |
| **μ=log(2/12)** | *1:1 CC* |  |  |  |  |  |  |  |  |  |
|  | **True expo** | 0.1 | 0.033 | 94.6 | 1.00 |  | 0.0 | 0.033 | 95.4 | 1.00 |
|  | **CC WTD** | -0.6 | 0.040 | 94.0 | 1.46 |  | -1.0 | 0.040 | 93.4 | 1.53 |
|  | **WTD prob** | 9.3 | 0.043 | 39.3 | 1.75 |  | 8.5 | 0.044 | 47.7 | 1.82 |
|  | **90 days** | -15.1 | 0.034 | 0.2 | 1.07 |  | -16.3 | 0.034 | 0.0 | 1.12 |
|  | **30 days** | -9.3 | 0.052 | 48.2 | 2.47 |  | -10.3 | 0.052 | 42.5 | 2.59 |
|  | *1:10 CC* |  |  |  |  |  |  |  |  |  |
|  | **True expo** | 0.0 | 0.040 | 94.3 | 1.00 |  | 0.0 | 0.040 | 94.8 | 1.00 |
|  | **CC WTD** | -0.3 | 0.046 | 95.3 | 1.27 |  | -0.4 | 0.046 | 95.1 | 1.32 |
|  | **WTD prob** | 7.3 | 0.052 | 65.7 | 1.65 |  | 6.5 | 0.053 | 72.8 | 1.71 |
|  | **90 days** | -12.6 | 0.043 | 11.4 | 1.14 |  | -14.0 | 0.044 | 5.5 | 1.18 |
|  | **30 days** | -13.9 | 0.060 | 27.3 | 2.21 |  | -14.6 | 0.061 | 25.4 | 2.32 |

*(*) Analysis methods:*

*1:1 CC* indicates 1 control per case, *1:10 CC* indicates 10 controls per case.

**True expo** – logistic regression with the actual exposure status as covariate (the reference analysis).

**CC WTD** – estimation based on joint likelihood for case-control status and the reverse WTD.

**WTD prob** – a reverse WTD is estimated to predict the probability of an individual being exposed and this exposure probability is used as covariate in logistic regression.

**90 days** – individuals are considered exposed if they have a redemption < 90 days before index date.

**30 days** – individuals are considered exposed if they have a redemption < 30 days before index date, logistic regression.

Table A3. Simulation results for the setting with data generated from a Weibull Backward Recurrence Density (BRD) and analyzed with a Log-Normal BRD. The datasets had a sample size of 19,800, and on average 80% of patients continue treatment at the index date, 25% of patients have a prescription redemption in the year before the index date and the true OR is 3. For each setting 2,500 datasets were generated and analyzed. The Weibull BRD used to generate data corresponded to a Weibull distribution with the same mean and variance as a Log-Normal distribution with $\mu$ and $\sigma$ as its parameters, see text for details.

|  |  | **σ=exp(-0.35)** | | | |  | **σ=exp(-0.25)** | | | |
| --- | --- | --- | --- | --- | --- | --- | --- | --- | --- | --- |
|  | **Analysis method** *(*)* | **Relative bias (%)** | **SE log(OR)** | **Coverage 95% CI (%)** | **VIF** |  | **Relative bias (%)** | **SE log(OR)** | **Coverage 95% CI (%)** | **VIF** |
| **μ=log(1.5/12)** | *1:1 CC* |  |  |  |  |  |  |  |  |  |
|  | **True expo** | 0.1 | 0.038 | 94.6 | 1.00 |  | 0.0 | 0.038 | 94.7 | 1.00 |
|  | **CC WTD** | 1.0 | 0.040 | 94.1 | 1.15 |  | 0.5 | 0.041 | 94.1 | 1.18 |
|  | **WTD prob** | 22.5 | 0.056 | 0.6 | 2.23 |  | 17.9 | 0.054 | 4.6 | 2.05 |
|  | **90 days** | -6.0 | 0.039 | 60.5 | 1.06 |  | -8.2 | 0.040 | 38.9 | 1.13 |
|  | **30 days** | -11.1 | 0.049 | 30.8 | 1.69 |  | -13.3 | 0.054 | 22.7 | 2.03 |
|  | *1:10 CC* |  |  |  |  |  |  |  |  |  |
|  | **True expo** | 0.1 | 0.052 | 95.7 | 1.00 |  | -0.1 | 0.052 | 95.4 | 1.00 |
|  | **CC WTD** | 0.7 | 0.054 | 95.3 | 1.08 |  | 0.5 | 0.054 | 95.2 | 1.10 |
|  | **WTD prob** | 11.3 | 0.069 | 56.1 | 1.78 |  | 8.4 | 0.067 | 72.2 | 1.68 |
|  | **90 days** | -6.4 | 0.053 | 73.8 | 1.04 |  | -9.7 | 0.054 | 50.9 | 1.09 |
|  | **30 days** | -16.7 | 0.062 | 15.3 | 1.45 |  | -19.8 | 0.067 | 8.3 | 1.71 |
|  |  |  |  |  |  |  |  |  |  |  |
| **μ=log(2/12)** | *1:1 CC* |  |  |  |  |  |  |  |  |  |
|  | **True expo** | 0.1 | 0.038 | 95.2 | 1.00 |  | 0.0 | 0.038 | 95.1 | 1.00 |
|  | **CC WTD** | 1.9 | 0.041 | 92.7 | 1.21 |  | 1.6 | 0.042 | 92.3 | 1.25 |
|  | **WTD prob** | 18.9 | 0.055 | 3.0 | 2.13 |  | 14.4 | 0.053 | 15.2 | 1.97 |
|  | **90 days** | -7.8 | 0.041 | 43.5 | 1.16 |  | -10.2 | 0.042 | 25.6 | 1.26 |
|  | **30 days** | -13.4 | 0.054 | 22.8 | 2.09 |  | -15.3 | 0.059 | 18.9 | 2.50 |
|  | *1:10 CC* |  |  |  |  |  |  |  |  |  |
|  | **True expo** | -0.1 | 0.051 | 95.0 | 1.00 |  | 0.0 | 0.051 | 94.6 | 1.00 |
|  | **CC WTD** | 1.4 | 0.054 | 94.7 | 1.12 |  | 1.6 | 0.055 | 93.4 | 1.14 |
|  | **WTD prob** | 8.5 | 0.067 | 71.8 | 1.71 |  | 5.7 | 0.066 | 84.3 | 1.63 |
|  | **90 days** | -9.8 | 0.054 | 48.9 | 1.10 |  | -12.9 | 0.056 | 28.2 | 1.17 |
|  | **30 days** | -20.0 | 0.068 | 9.0 | 1.74 |  | -22.4 | 0.074 | 7.5 | 2.06 |

*(*) Analysis methods:*

*1:1 CC* indicates 1 control per case, *1:10 CC* indicates 10 controls per case.

**True expo** – logistic regression with the actual exposure status as covariate (the reference analysis).

**CC WTD** – estimation based on joint likelihood for case-control status and the reverse WTD.

**WTD prob** – a reverse WTD is estimated to predict the probability of an individual being exposed and this exposure probability is used as covariate in logistic regression.

**90 days** – individuals are considered exposed if they have a redemption < 90 days before index date.

**30 days** – individuals are considered exposed if they have a redemption < 30 days before index date, logistic regression.

Table A4. Characteristics of cases and controls in empirical application. Cases with severe upper gastrointestinal bleeding in Funen County 1999-2006, with their controls, matched by sex and birthyear.

|  | **Cases**  **n= 3,568** | **Controls**  **n=35,552** |
| --- | --- | --- |
| *Demographics* |  |  |
| Age, median (IQR) | 75 (64-83) | 75 (64-83) |
| Male sex | 1,811 (50.7%) | 18,029 (50.7%) |
| *Current drug use* |  |  |
| VKA | 183 (5.1%) | 823 (2.3%) |
| ASA | 696 (19.5%) | 3,436 (9.7%) |
| Other antiplatelet drugs | 197 (5.5%) | 782 (2.2%) |
| SSRI | 429 (12.0%) | 2,038 (5.7%) |
| Systemic corticosteroids | 384 (10.8%) | 1,638 (4.6%) |
| PPI | 521 (14.6%) | 2,037 (5.7%) |
| H2 receptor antagonists | 294 (8.2%) | 958 (2.7%) |
| Statins | 237 (6.6%) | 1,572 (4.4%) |
| Nitrates | 318 (8.9%) | 1,678 (4.7%) |
| Spironolactone | 208 (5.8%) | 599 (1.7%) |
| Calcium antagonists | 588 (16.5%) | 3,829 (10.8%) |
| Bisphosphonates | 70 (2.0%) | 439 (1.2%) |
| *History of* |  |  |
| UGIB | 95 (2.7%) | 175 (0.5%) |
| HP eradication | 160 (4.5%) | 467 (1.3%) |
| Peptic ulcer | 218 (6.1%) | 535 (1.5%) |
| COPD | 256 (7.2%) | 1,044 (2.9%) |
| Diabetes | 404 (11.3%) | 2,167 (6.1%) |
| Ischemic heart disease | 867 (24.3%) | 5,272 (14.8%) |
| Heart failure | 279 (7.8%) | 1,164 (3.3%) |
| Stroke | 353 (9.9%) | 1,835 (5.2%) |
| Hypertension | 412 (11.5%) | 1,863 (5.2%) |
| Inflammatory bowel disease | 23 (0.6%) | 107 (0.3%) |
| Malignant disease | 244 (6.8%) | 1,711 (4.8%) |
| Renal failure | 94 (2.6%) | 205 (0.6%) |
| Alcohol-related markers | 166 (4.6%) | 336 (0.9%) |
| Tobacco-related markers | 1,148 (32.1%) | 8,364 (23.5%) |
